# Farmers’ and Experts’ Knowledge Coping with Sheep Health, Control and Anthelmintic Resistance of Their Gastrointestinal Nematodes

**DOI:** 10.3390/pathogens13040297

**Published:** 2024-04-02

**Authors:** Jacques Cabaret, Christian Nicourt

**Affiliations:** 1 INRAE and University F. Rabelais, ISP UMR 1282, 37380 Nouzilly, France; 2 INRAE, CNRS and University Paris-Dauphine, IRISSO, Place Maréchal de Lattre de Tassigny, 75775 Paris CEDEX 16, France

**Keywords:** farmers’ knowledge, experts, sheep, disease, gastrointestinal nematodes, anthelmintic resistance

## Abstract

Gastrointestinal nematodes are common in grazing sheep, but the intensity of the infection is not easily appreciated by farmers. For decades, they have relied on anthelmintic treatments to control these gastrointestinal nematodes. This has led to anthelmintic resistance and poor control of infection in most regions of the world. Using face-to-face semidirective interviews with farmers, we recorded their views on gastrointestinal nematode infection and its control. Ten organic and nine conventional meat sheep farmers from central France and 20 milk sheep farmers from the Basque region were interviewed and the data were analysed using a health model based on the importance of the disease and the barriers to implementing control. It was found that gastrointestinal nematodes were not the main health concern, and therefore farmers were not willing to invest too much time and money in their control. The conventional farmers relied on their veterinarians (the experts) to organise and select the anthelmintics, although they complained about the limited investment of their veterinarians in this matter. Organic farmers complained about their lack of knowledge about complementary and alternative medicines. Farmers rarely used faecal egg counts to build control of gastrointestinal nematodes and were unaware of the intensity of their infection. Knowledge of anthelmintic resistance was general (it exists) but farmers did not know if it existed on their farm. Resistance was often considered to have come from elsewhere, so the farmer did not feel at fault and did not take measures to prevent resistance. There is a need for all stakeholders to use faecal egg counts to assess the intensity of infection as well as the level of anthelmintic resistance to establish individual farm control programmes rather than standard blanket treatments.

## 1. Introduction

Sheep rearing in Europe occurs mostly in disadvantaged agricultural areas, where grazing animals on pastureland is often the only way to add economic value [1]. In France, 95% of the ruminants are grazed on pastures [2]. It is a subsidized husbandry (up to 165% of the income before taxes) by the CAP (Common Agricultural Policy) of the European Union [3]. Gastrointestinal nematodes (GINs) infect all ruminants grazing on pasture. The life cycle of a GIN consists of two successive stages, one with free-living infective larvae on grass, and the other as a parasite of the digestive tract of ruminants that ingest the larvae. Infection may be associated with symptoms such as diarrhoea (*Trichostrongylus*) or anaemia (*Haemonchus*) in heavy infections, but more often there are no specific symptoms indicative of the disease. In most cases, infection is associated with a reduction in production (milk or meat). Diagnosis of the infection is easily achieved by examination of ruminant faecal samples, in which GIN eggs are detected. The use of laboratory diagnosis is not common [4,5,6], and farmers mostly rely on the use of anthelmintics without this laboratory test [7]. Their assessment of the infection is not accurate, as shown in sheep [8], or in dairy goats [9], where there was no correlation between actual and estimated infection, and, thus, GIN infection is often a camouflage disease not detected by farmers. The repeated use of anthelmintics over the years has led to the emergence of GIN-resistant populations [10]. We are then faced with a situation where the disease may be undetected, and treatments are often ineffective. In such a complicated situation, it is not surprising that farmers can build their knowledge from very different sources (learning from books, journals, internet, own experience, parents, neighbours, other farmers or technicians, and veterinarians [4,11,12] and follow different strategies not related to the actual situation of GIN infection. The knowledge of the experts (here, veterinarians or technicians) is very different: it is based on biological knowledge and pretends to be universal; the guidelines for dealing with GINs are almost the same everywhere [13]. This is slowly changing, for example, in Australia, where local recommendations are proposed [14]. Farmers’ knowledge is rooted in local conditions and farmers’ beliefs [14]. Farmers’ knowledge is also partly tacit [15]. Veterinarians are often unable to identify farmers’ main goals [16] and may benefit from tailoring their advisory services to farmers’ specific worldviews [17]. This may explain why compliance with veterinary advice is not strictly adhered to [11,13,18]. Thus, the expert role of the veterinarian can be questioned in sheep farming [12,19] and especially in organic farming [20]. The changes in veterinary recommendations have also been very dramatic: from treating the whole flock and moving them to a clean pasture [21] to treating only those animals with GIN problems (targeted selective treatments) when they are still on an infected pasture [22]. The idea of treating only those animals in need of treatment (selective treatment) rather than the whole herd was probably inspired by Anderson and Medley [23] from the modelling of infection by a human nematode (Ascaris), which was later applied in the field to reduce the cost of treatment without any adverse effect on infection intensity [24]. These changes in veterinary strategies to control GINs were not due to a reduction in the cost of treatment, but to the spread of anthelmintic resistance. The idea of the researchers was to reduce the selective pressure for GIN resistance while maintaining an acceptable level of infection compatible with meat or milk production [25]. These changes in veterinary strategies for GIN control may also change positive attitudes towards the veterinary profession: what was good in the past is no longer relevant, so what is the good strategy? Farmers are also confronted with a range of nonsynthetic treatments derived from plants, which are not considered anthelmintics, but which promote a reduction in the side effects of GIN infection. Some veterinarians, husbandry technicians, or farmers may promote the use of these treatments and then be considered as experts by organic farmers (but not only) [26]: it is then a situation of multiple expertise. There is a gap between veterinarians’ recommendations and farmers’ practices, so there is a need to understand farmers’ views on GINs. We will use Abraham and Sheeran’s [27] Health Belief Model. The model focuses on individuals’ representations of threat perception and behaviour evaluation. These representations are shaped by the farmer’s values (choice of conventional or organic husbandry), type of production (meat or milk), and his personal (family and neighbours) and technical environment (peers, technicians, and veterinarians). Threat perceptions are based on perceived susceptibility to disease and the expected severity of the consequences of disease. This notion of threat is highly important, since farmers are confronted by many challenges, sheep health being one of them, and with GINs being a part of health control. We will focus on GINs in a larger context of sanitary problems. Behavioural evaluation consists of beliefs about the benefits of a recommended health behaviour and beliefs about the barriers to performing the behaviour. Farmers will have to assess the threat of GINs (risk of infection to their sheep) and the consequences for production (meat or milk) or health (diarrhoea or anaemia). The benefits of the treatments will be weighed against the costs and work involved in administering them. Estimating the threat of anthelmintic resistance is more difficult without laboratory control of infection before and after treatment [26,28] and is, therefore, not fully estimated by farmers [6,29,30]. Farmers’ actions to reduce anthelmintic resistance are likely to be limited. Since farmers’ knowledge is rooted in local conditions and their own views, it is useful to compare different localisations among sheep production regions and management practices (milk or meat, conventional or organic rearing). All information was based on farmers’ declarations and represented their opinions on health with a focus on gastrointestinal nematodes control and resistance.

## 2. Materials and Methods

### 2.1. Description of the Farms (Table 1)

Central France (Massif Central) is a region of mostly plains and semimountains (less than 1200 m above sea level). The average rainfall is 850–1000 mm and the average temperature is 10.9–11.4 °C. Meat sheep production is a major production of the region, either under conventional or organic management [2,4]. The size of organic farms is smaller than that of conventional farms, mostly due to farmers with a nonagricultural background who have entered the profession, while conventional farmers may have inherited all or part of their land. The organic farms were mostly located in the Montluçon area based on previous contact (presence of a local advisor with interest and competence in organic farming); the conventional farms were located between Poitiers and Limoges. In the Basque region (in the southwest of France, near the Pyrenees mountains: Basse-Navarre and Soule), the farms have historically had a small area of agricultural land [31] and rely on communal or mountain pastures [32]. The average rainfall is 1248–1312 mm and the average temperature is 13.2–14.3 °C. They breed dairy ewes and 60% also produce high-quality cheese, Ossau-Iraty. The ewes are maintained in a sheepfold during winter. The professional farmers selected had at least five years’ experience and were among the most involved in improving their management. The conventional farmers in central France were mostly of rural origin, whereas the organic farmers were not. The Basque farmers were also often of nonrural origin.

**Table 1:** Description of farms and farmers investigated.

Location	Main Production	Organization (Number of Farms)	Average Flock Size (Ewes) and Area of the Farm (ha)	Farmer’s Age and Gender	Rural Origin and Initial Education in Agriculture (% Farms)
Central France	Meat sheep	Conventional (9)	665 (173) * 150 (65)	47 (10) * 100% male	70 80
		Organic (10)	467 (229) 91 (35)	50 (10) 100% male	30 30
Basque Region	Dairy sheep	Conventional (20)	249 (117) 20 (16)	48 (11) 85% male	45 85

* Average and standard deviation.

### 2.2. Farmers Semidirective Interviews

The interviewers (J.C. and N.C.) asked the farmers open questions, as described by Hughes [33]. These questions had been preprepared in an interview guide, which was identical for all interviews (Supplementary Data S1). The interview guide for the dairy plant, chambers of agriculture, and cooperative technicians was adapted from the farmers’ guide. The recorded interviews were transcribed into a Word text. All the texts from one group of farmers were concatenated before analysis. Tropes (V8.5) [34] speech/discourse analysis software was first used to process the data for the cognitive analysis of the interviews [35], which were then analysed using the multivariate method [36] applied to the most frequently used words in the interview. The difference between discourse analysis in our study and text linguistics is that discourse analysis aims at revealing sociopsychological characteristics of a farmer rather than this text structure. Significant differences in the word’s occurrences between farming types were assessed using two-tailed Z score statistics for two populations; where the proportions were low (less than 4%), Fisher’s exact test was applied to the number of occurrences for each word. Technical questions on GIN management (type of anthelmintic treatment, frequency, and pasture use) were asked separately when they were not spontaneously provided during the interview. We built a grid and codes from manually analysing farmers’ speech: importance of strongyle infection; number of treatments; use of selective treatments, of natural (mostly plants or essential oils of plants) or synthetic anthelmintics; choice of anthelmintics; Is there a visible effect of the treatment? Is the veterinarian an important advisor in relation to GINs? Is resistance to anthelmintics present in the farm? Is it imported from elsewhere? According to the speech and answers of the farmers, we translated the information into semiquantitative variables. We coded these variables from one (low) to three (high). For example, quantitative data like the number of treatment were coded as follows: 1—no treatment, 2—once/twice a year, 3—more than twice a year. Qualitative data like the use of natural or synthetic anthelmintic were coded as follows: 1—natural, 2—both, 3—synthetic only. The variables were subjected to centroid cluster analysis using nonparametric Spearman correlation coefficients. The centroid analysis used average linkage, which was a balanced approach to clustering. The centroid of each group was calculated and the distance between the groups was the distance between centroids.

## 3. Results

### 3.1. The Farmers’ World as Seen in the Difference of Words’ Occurrences (Table 2)

Sheep was the most used word, used followed by farmer (58.1% of the word occurrences in the Basque region) and veterinarian (13.3% in the conventional farms of central France), which is not surprising since the interviews were related to sanitary problems. The role of farmer was apparently more important (organic central France and Basque region) than the one of the veterinarians, or equal (conventional farms from central France). The presence of technician, parent, or peer/neighbour was also acknowledged by all farms. The word parent was most used among conventional farmers of central France in relation to their rural origin (proximity and inheritance of farms from parents). Shepherd (21.1% of occurrences) and mountain (17.8% of occurrences) were highly present for the farmers of Basque region due to the use of transhumance towards Pyrenees mountains and the necessity of shepherds to manage the flock on extensive pastures. The word parasite and treatment were frequent in the interviews of organic farmers because they first used alternatives, and then synthetic anthelmintics. 

**Table 2:** The percent occurrence of words used by different categories of farmers.

Words Employed (In Percent of the Most Used Word-Sheep)	Central France (Organic) a	Central France (Conventional) b	Basque Region (Conventional) c	Significance (Chi-Square or Exact Fisher Test)
Farmer	30.2	14.0	58.1	c > a > b
Veterinarian	7.0	13.3	4.7	b > a > c
Parent	7.0	14.5	2.5	b > a > c
Neighbour/Peer	6.4	4.7	5.1	a > b = c
Technician	7.4	4.3	4.8	a > b = c
Shepherd	0	0	21.1	c > a = b
Mountain	1.8	0	17.8	c > a > b
Parasite	6.4	0.1	1.5	a > b = c
Treatment	16.1	10.1	3.8	a > b > c

### 3.2. Sanitary Problems According to Farmers

The main sanitary problems listed by farmers are presented in Table 3.

The central France farms’ words of pathology looked alike, either organic or conventional (Spearman rho 0.81, *p* = 0.001), but were different to Basque region farms (respectively, Spearman rho −0.41 and −0.21, *p* > 0.05). Parasites were considered as more important in farms from central France where there is production of lambs rather than in farms of the Basque region, where milk production and, hence, ewes were the focus of husbandry. Among the latter, flukes were not mentioned, and major problems were contagious agalactia, mastitis, and footrot. Gastrointestinal nematodes importance was decreasing from organic, conventional farms of central France to Basque region farms. Faecal egg counts were not frequently cited (0.5, 0.4, and 0.3 respectively).

### 3.3. Multivariate Farmer’s and Veterinary Worlds

These worlds are centred on the farmer or the veterinarian, based on interviews with farmers. Farmer or veterinarian is the word we selected to be in the centre of the figure and then it is related with other words that occur jointly and frequently. The farmer figure is what he thinks about him and his work as a sheep farmer, and in the veterinarian figure it is how he relates the veterinarian to his own sheep husbandry.

**Figure 1:**
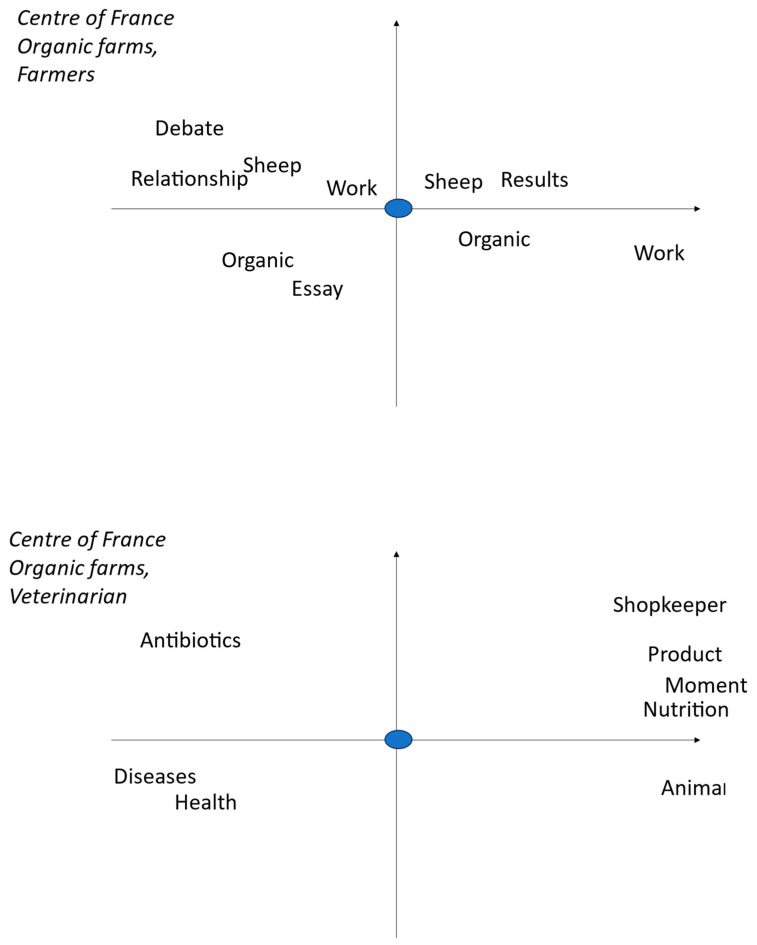
Multivariate analysis of farmers interviews with tTropes in organic farms of central France: vision of themselves and how they see the veterinarians (blue dot). The left part of the graph corresponds to the beginning and the right part to the ongoing interview. The words are exactly located on the figure at the place of their first letter. Words in the same location on the figure are related. The words near the blue dot are strongly related to the farmer or veterinarians and those far away are only loosely related.

The views of organic farmers from central France are (Figure 1):

- Working with sheep is the main concern;
- Being and staying organic is important;
- This is achieved through essays and information with others.

For them, the veterinarian is in charge of health and diseases; he is needed at different moments, but he mainly gives therapies (product and antibiotics) and he is considered as a kind of shopkeeper.

**Figure 2:**
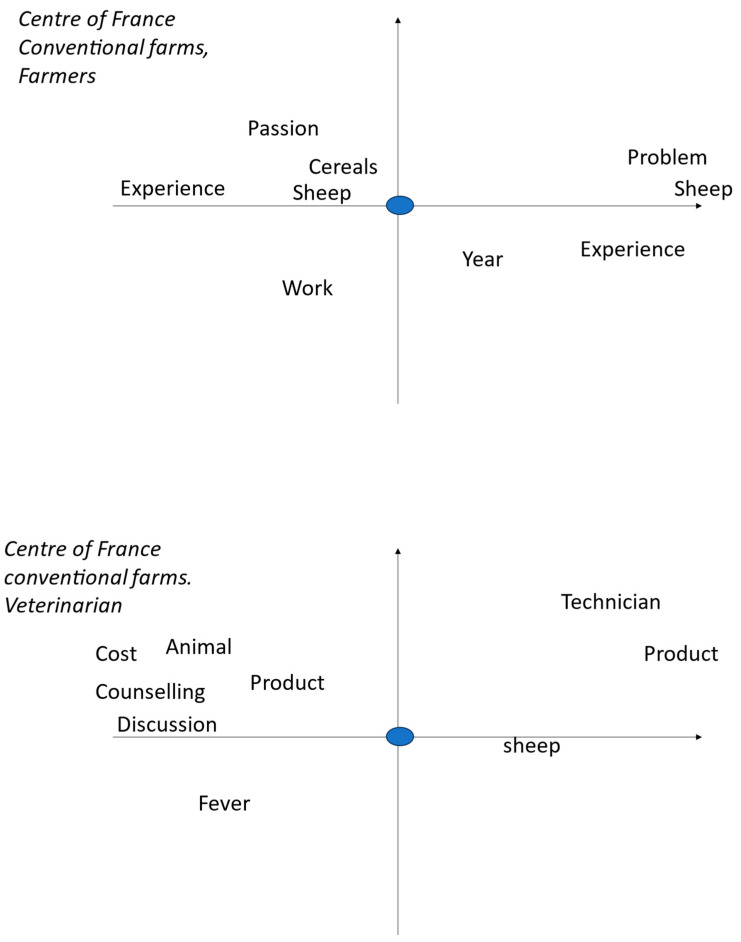
Multivariate analysis of farmers interviews with tTropes in conventional farms of central France: vision of themselves and how they see the veterinarians (blue dot). The left part of the graph corresponds to the beginning and the right part to the ongoing interview. The words are exactly located on the figure at the place of their first letter. Words in the same location on the figure are related. The words near the blue dot are strongly related to the farmer or veterinarians and those far away are only loosely related.

The views of conventional farmers in central France are (Figure 2):

- Sheep and cereal production are the main concerns;
- Passion is needed because the work is difficult and requires experience;
- Variations from one year to another (problems with sheep).

For the farmers, the veterinarian is expensive, but provides information and advice in case of symptoms (fever) and provides the necessary medicine (product). It should be noted that the role of technicians is also recognised.

**Figure 3:**
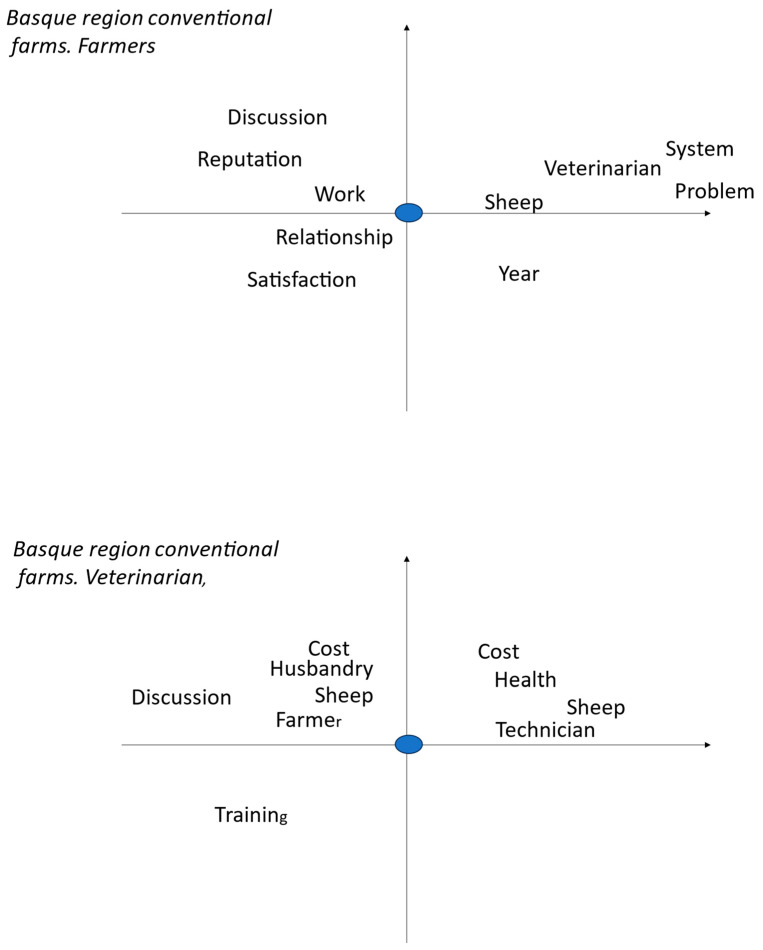
Multivariate analysis of farmers interviews with tTropes in conventional farms of the Basque region of France: vision of themselves and how they see the veterinarians (blue dot). The left part of the graph corresponds to the beginning and the right part to the ongoing interview. The words are exactly located on the figure at the place of their first letter. Words in the same location on the figure are related. The words near the blue dot are strongly related to the farmer or veterinarians and those far away are only loosely related.

The views of farmers from the Basque region are (Figure 3):

- Work is a major concern in terms of discussion, reputation, and relationship with others, and, finally, should bring satisfaction;
- Sheep is associated with veterinarian and problem, system of breeding, and with variations between years.

### 3.4. Gastrointestinal Infection, Anthelmintic Resistance, and Role of Veterinarian According to Farmers

The following words/sentences were extracted by us from interviews and from additional questions: GIN, anthelmintic resistance is present on farm, it originated from elsewhere, number of treatments with synthetic anthelmintics, choice of anthelmintics, use of natural treatments, use of selective anthelmintic treatment, visible results of these treatments, importance of the veterinarian in control. The information was gathered from the centroid cluster analyses (Supplementary Figure S1). The GIN intensity of infection, the existence of resistance, and the role of the veterinarian were differently associated among the three groups of farmers. 

GIN infection was negatively related to the number of anthelmintic treatments in central France and positively related to the limited choice of anthelmintics in lactating ewes in the Basque region. The role of resistance was not consistent among the three types of farmers, either positively (Basque region) or negatively (central France) associated with GIN infection. The veterinarian was associated with the choice of anthelmintics in all farms, although it was considered either positive or negative for the results on GIN infection or resistance. The promotion of anthelmintic treatments was rated differently: positively on GIN infection, and negatively on anthelmintic resistance.

## 4. Discussion

Farmers involved in husbandry are confronted by many problems to solve depending on the structure of the farm and size of flock, their production, and their geographic location (see the occurrence of the word mountain for Basque region in Table 2). The size of farm in ha is important in a subsidised agriculture like in Europe; the size of farm is also related to the size of flocks. They were much larger in our farm samples (Table 1) than the European average of 113 ewes (1), and the flock size in central France was like the European highest value in Western Wales (UK). Central France is rather dedicated to meat sheep and represents 21% of the French production, and the Basque region harbours 60% of the dairy ewes (2). The studied farms are, thus, quite representative of the main regions of sheep production in France and are among the largest in Europe. 

The farmers’ world is a key factor to understand their management and their behaviour in handling complex problems [37]. There are striking differences among the three types of farms (Table 2): the organic in central France and the conventional ones of Basque region place much importance on the person(s) involved in the daily care of sheep, the farmer or the farmer and the shepherd, respectively. The central France conventional farmers very often cite the veterinarian (who provides them blanket treatments) and the parents (from whom they often inherited the farm) (Table 2) and probably minimize their role. This means that the advisory strategy will have to be different according to the type of farmers: it should be the farmer itself in organic farms of central France, the farmer and the shepherd (when different) in farms of the Basque region, and, finally, several persons (the farmer, the vet, and the family) in conventional ones in central France. The farmers viewed the veterinarian as a major actor in relation to animal health [38], but organic ones from central France liked to have their own essays with different treatment providers and considered the veterinarian as a shopkeeper delivering drugs (Figure 1), as already seen by [20], whereas their conventional colleagues complained about the cost of veterinarians but admit to having discussions and receiving advice from them, in addition to obtaining the needed drugs (Figure 2). The farmers of the Basque region also complained about the cost of veterinarian, the lack of interest or expertise for sheep for some of them, as noted in the UK [19], even if they still regard them as specialists of animal health; they also acknowledged the role of the technicians (Figure 3).

The central France farms’ words of pathology looked alike, either organic or conventional, but differed from those of the Basque region. In this region, in addition to the presence of contagious agalactia [39], there were more mentions of footrot, mastitis, and diarrhoea. There were fewer mentions of parasitosis in general and, in particular, of coccidiosis, myasis, and gastrointestinal nematodes, and none of fasciolosis and paramphistomosis (Table 3). These two helminths are highly prevalent in central France [40] but are rare in Basque regions, less than 7% of ewes [41]. Farmers do have several sanitary problems and GINs are only one of them, so the control of GINs is to be understood within this environment. Farmers know that GINs are present on their farms and that they are detrimental to their sheep. They can assess the performance of the lambs in terms of weight gain but are unable to assess GIN infection [8]. They can also assess milk production in lactating ewes, but this depends on many factors and varies from day to day, so it is not easy to relate it to GIN infection. The actual infection is seldom evaluated by faecal nematode egg counts in the laboratory in our studied farms, and it is not often used in other European situations, like in Ireland [6]. It is practised by Basque organic farmers when they want to justify the use of synthetic anthelmintics instead of natural ones based on plants [38]. The use of faecal egg counts is Australia is more frequent (up to three times a year [39]), possibly due to inactivity of many anthelmintics for many years, backward compared to in Europe, and to the availability of kits to conduct these faecal egg counts on farms. The very limited use of faecal egg counts, in our conditions, is a major problem to build a rational estimation of threat. In addition to the time and cost of laboratory faecal egg counts, we noticed that the farmers were often deceived by the laboratory results, since they did not know how to interpret them [4]. The nematode fauna central France [40] is characterised by the presence of *Trichostrongylus axei* alongside the dominant species recorded in other areas (*Teladorsagia circumcincta* and *Trichostrongylus colubriformis* with few *Haemonchus contortus* and *Nematodirus*); therefore, there was no simple indicator of their presence, such as the FAMACHA© anaemia score [41], which is often related to *H. contortus* infection. Similarly, the nematode fauna in the Basque region is mainly composed of *T. circumcincta*, *T. axei*, and *Cooperia* in equal proportions, and *T. colubriformis*, *Nematodirus*, and *H. contortus* in small proportions [42], and does not also support the use of the anaemia score. Farmers are confronted with many other diseases, and therefore consider GINs as one of their threats, but contagious agalactia in the Basque region [43] or flukes in central France [44] are more important concerns (Table 3). All farmers relied on veterinarians for health problems (including GINs) in terms of information and treatment (Figure 1), although they were not fully satisfied with their involvement in the Basque region [38], as seen in other places [16,20]. Organic farmers even questioned their competence in complementary and alternative medicines, as already mentioned elsewhere [20,26,38]. Farmers’ behaviour towards GINs does not follow the information deficit model, according to which the informed adopt the correct opinion when provided with sufficient information, as already shown in other health problems [45]. Farmers know about GINs from journals, peers, technicians, and veterinarians, and that the only way to assess GIN infection is by laboratory faecal egg counts [4], but they do not use them frequently. It is rather due to cognitive miser, a tendency to think and solve (apparently minor) problems in the simplest and least effortful way [46]. This tendency is exacerbated by the heavy workload of farmers (Figure 1) and the complexity of GIN management.

Farmers’ views on anthelmintic resistance are unclear. Anthelmintic resistance is common in Europe [10], and central France [47,48] or the Basque region are no exception [49]. Farmers’ knowledge of and commitment to the issue of anthelmintic resistance is low, as has also been reported in cattle [30] and sheep [50], although their involvement is necessary to maintain control of GINs [29]. Anthelmintic resistance could not be associated with any factor among organic and conventional farmers in central France (Table 4), indicating either disinterest or lack of knowledge about anthelmintic resistance. It was not a lack of interest since it was perceived as a factor involved in the intensity of GIN infection, probably because of the poor efficacy of the drugs. Farmers did not deny the existence of anthelmintic resistance in general, but they were not too concerned themselves, since for most of them, the resistance originated elsewhere. Consequently, they were not at fault and did not need to change their practices. In cattle, the knowledge about anthelmintic resistance did not promote the adoption of faecal egg counts or other diagnosis of GINs [51]. In Scottish sheep, the adoption of best practice in GIN control (quarantine prior to introduction and testing for infection with faecal egg counts, among others) was shown to be related to the expectation of others (quarantine) and the belief that there might be anthelmintic resistance on the farm and, hence, laboratory testing and greater use of faecal egg counts [12]. The reality of on-farm resistance was not established in our study. This apparent lack of concern may explain why resistance to anthelmintics was not consistently appreciated by different groups of farmers. 

The barriers to implementing good practices to control GINs were the following: (1) GINs are not the main sanitary problem; (2) the anthelmintics have proven efficient for decades and were the unique solution for control; (3) workload is high and it is not easy to implement new measures; (4) the veterinarian is consulted more for acute problems (a firefighter, as in [19]) than for the GIN control strategy. Farmers’ views should be known before implementing good practices, and veterinarians should be involved in more than advising and providing anthelmintics. These conclusions on sanitary problems are based on farmers’ opinions and do not always reflect reality. Nevertheless, veterinarians seeking to positively engage farmers in advisory interactions could consider focusing on farmers’ motivations and goals as paramount in framing advisory messages [17,37]. Engaging all stakeholders (veterinarians, farmers, and technicians) could be a promising way to generate knowledge and rational decision making about GIN management.

## Figures and Tables

**Table 3:** The occurrence of words related to pathology per farms among different categories of farmers.

Words Employed (Occurrence/Number of Farms)	Central France (Organic))	Central France (Conventional)	Basque Region (Conventional)
Diseases			
Contagious Agalactia	0	0	5.5
Parasitosis	5.4	4.3	1.4
Fasciolosis	4.2	3.9	0.1
Paramphistomosis	0.7	0.6	0
Gastrointestinal nematodes	1.6	0.6	0.2
Moniezia	0.9	0.8	0.6
Coccidiosis	0.7	0.6	0
Myasis	1.2	2.0	0.2
Scabies	0.6	1.1	0.5
Footrot	0.5	0.6	1.3
Tetanos	0.4	1.1	1.3
Symptoms/syndromes			
Abortion	1.8	2.1	0
Fever	0.8	1.6	0.5
Coughing	1.4	0.9	0
Diarrhoea	0.5	0.9	2.2
Mastitis	0.2	1.0	1.9

**Table 4:** Opinions of farmers on gastrointestinal nematodes (GINs), resistance to anthelmintics, and role of the veterinarian.

	Variables or Groups of Variables in Relation with the Parameter		
Parameters	Central France (Organic)	Central France (Conventional)	Basque Region (Conventional)
GIN intensity of infection	Natural treatments, visible effect of treatments. (0.4). Veterinarian, no. of treatments, resistance from elsewhere *(−0.6).*	Resistance is coming from elsewhere, use of natural treatments (0.7) *. Resistance exists, visible results, selective treatments, no. of treatments *(−0.6).*	Resistance exists, Choice of anthelmintics *(0.5).*
Resistance of GINs to anthelmintics exists	None (−0.2).	Number of treatments, choice of anthelmintics, veterinarian, resistance is coming from elsewhere (0.4).	Choice of anthelmintics, intensity of GIN infection *(0.5).*
Role of veterinarian	No of treatments *(0.9).* Choice of anthelmintics, resistance exists, natural treatments, visible results, GIN intensity of infection. *(−0.6).*	Choice of anthelmintics *(0.6).* Resistance exists, visible results, selective treatments, no. of treatments *(−0.6).*	Choice of anthelmintics, intensity of GIN infection, resistance exists *(0.5).*

* Spearman coefficient of correlation, in italics when *p* < 0.05.

## Data Availability

Data are contained within the article.

## Supplementary Materials

The following supporting information can be downloaded at: https://www.mdpi.com/article/10.3390/pathogens13040297/s1, Figure S1: Centroid cluster analysis of the views of farmers on gastrointestinal nematodes infection and control in three types of farms; Data S1: Guide for interviews on the health experiences and strategies of sheep farmers.
