# Supplementary material for: Farmers’ and Experts’ Knowledge Coping with Sheep Health, Control and Anthelmintic Resistance of Their Gastrointestinal Nematodes
Source: Pathogens. 2024 Apr 2;13(4):297. doi: 10.3390/pathogens13040297 (PMC11054267; doi:10.3390/pathogens13040297)
Supplement: Supplementary file 1 [file pathogens-13-00297-s001.zip › pathogens-2885416-supplementary-Data S1.pdf]

GUIDE FOR INTERVIEWS ON THE HEALTH EXPERIENCES AND STRATEGIES OF SHEEP FARMERS.

1- Personal and professional background

Life history of the breeder and entering his profession

How did you learn husbandry? What have you learned since setting up in your farm that you didn't know before?

Can you describe the problems you encountered in your early years?

How has your profession and your farm evolved? And around you?

What are the difficulties of being a sheep farmer?

(why did you opt for organic farming; what are the difficulties of conversion?)- optional.

2- The sheep farming profession

What are the differences between sheep farmers?

Diversification within the profession: processing, sales, etc.

Does it mean that you have several professions being a sheep farmer: which ones?

Organic/non-organic: what are the differences in the way the profession is conceived and practised? - optional.

How does the profession evolve in your farm? What are the transitions?

3- Work on the farm and its organisation

The current technical characteristics of the farm: number of animals, surface area, food self-sufficiency, processing, sales, etc.

How is your farm organised? How did you arrive at this organisation? Who does what?

Can you describe your work last week?

4- What collective activities?

Technical exchanges within the farm

Outside the farm: organisations such as sanitary groups, husbandry groups, agricultural materials in common, peers, neighbours, technicians, vets, etc.

Who are your closest colleagues? Do you see each other often, what do you do together?

With whom do you discuss your work, are you in technical discussion groups?

5- How is work judged in sheep farming?

What is a good farm? What are beautiful ewes or flock?

What is a good work for a sheep farmer? And the dirty work?

6- What is your health strategy on your farm?

What is care work?

How do you monitor health?

Prevention: introductions, quarantine, genetics, rotational grazing, etc.

How does the problem of care arise on your farm?

What makes your farm different from others in terms of health?

The biggest health problems you've had, how did you solve them? Who did you rely on: colleagues, technicians (which ones), vet... What was missing at the time; what posed a problem?

From a health point of view, what is the day-to-day work?

When and what do you consider to be doing your job well, because you have the time?

What do you consider to be doing a dirty work, may be because you're in a hurry?

What is a normal health situation in your farm?

How do sheep genetics influence the health situation?

What is a hardy animal?

7- How do health problems manifest themselves?

What are the first indicators?

Do you have "sentinel" animals?

Are there any risk situations on your farm?

What are the health histories on your farm: flock, individual animals?

Do you use the expression "cruising flock", flock in equilibrium?

What is a health crisis: what are the thresholds, the transition from an individual to a batch, an epidemic, etc.?

What does it mean to work in a crisis situation? What happens, who do you see, who can you count on?

What impact do crisis situations have on the work: experiences, relationships with colleagues?

What is the situation regarding scrapie and infectious anaemia, parasites- Basque region, bluetongue, parasites- Central France?

8- Which health treatments?

(Is organic livestock farming helpless in the face of health problems?)- optional.

(organic: how do you deal with health issues without treatments?)- optional.

What do you mostly use: individual treatments/treatment of batches?

What phytopharmaceutical products do you use?

Phytotherapy, essential oils, homeopathy, etc.?

Why did you choose them: how, with whose help, and why did you choose them to continue afterwards?

How do you treat the animals: injections, drinking, incorporating feed, manual applications, other? Use of foot baths?

Do you disinfect your sheep fold, other buildings; who does this work?

9- How did you build up your health experience, on what occasions, with whose help?

Which colleagues helped you and how?

Which technicians?

Which vets?

How do the sheep help you identify their health problems?

How does the flock help you identify sick animals?

How does the sick animal help you spot it?

How do the animals contribute to their care (by calling on the breeder)?

How do animals contribute to their care by self-medicating: like cats purge themselves?

10- How do you write your working methods and results?

Do you have a set of specifications?

How is this a constraint and how can it help in terms of health?

How do you manage your breeding data: notes in a notebook, in a computer/tablet, do you use technical and economic management tools?

<u>11- How are the ways of working in sheep farming and the profession changing?</u>

Additional questionnaire used when we did not obtain information on parasites and treatments:

Do you have problems with digestive-tract strongyles?

Do you have faecal egg counts performed?

Do you treat your sheep with anthelmintics?

How? And which ones?

Based on vet advice?

Have you heard about anthelmintic resistance?
