# Supplementary material for: Farmers’ and Experts’ Knowledge Coping with Sheep Health, Control and Anthelmintic Resistance of Their Gastrointestinal Nematodes
Source: Pathogens. 2024 Apr 2;13(4):297. doi: 10.3390/pathogens13040297 (PMC11054267; doi:10.3390/pathogens13040297)

Figure S1: Centroid cluster analysis of the views of farmers on gastrointestinal nematodes infection and control in three types of farms

Codes of semi-qualitative data:

Importance of strongyles-GIN: strii

Resistance originated from elsewhere: reai

Resistance exists on farm: rese

Number of treatments with synthetic anthelmintics: trant

Choice of anthelmintics: cant

Use of natural treatments: tsna

Use of selective treatments: tsel

Visible results of the treatments: revi

Importance of the veterinarian: veto

Conventional meat sheep (Centre of France)

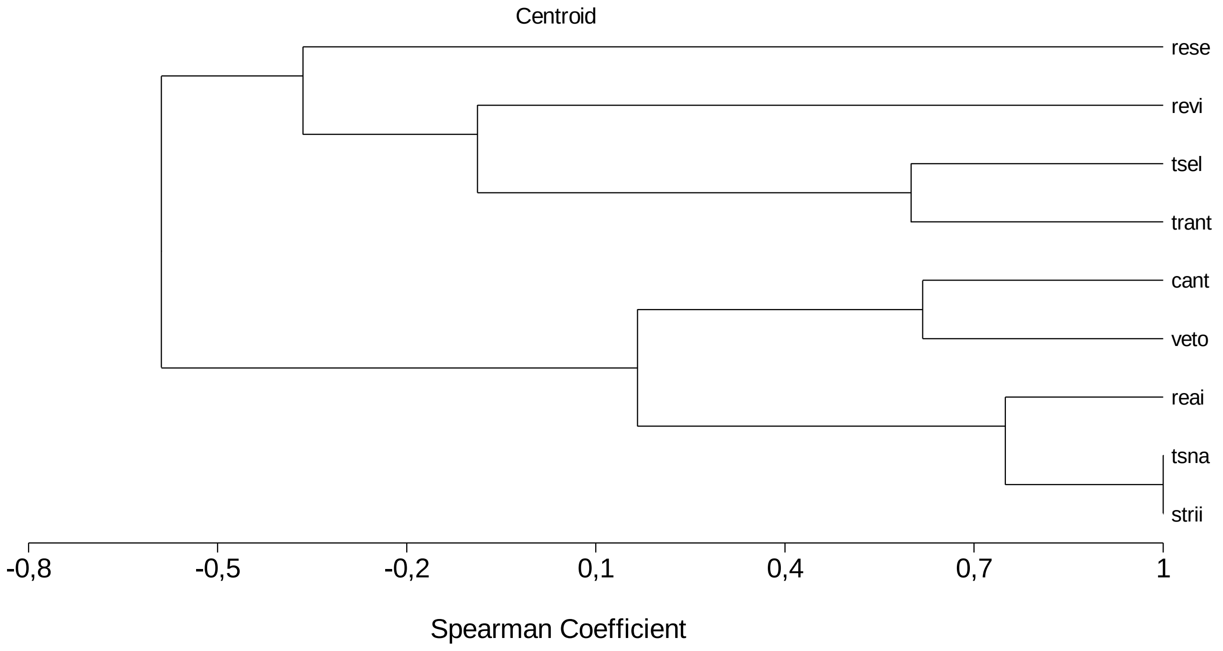

Organic meat sheep (Centre of France)

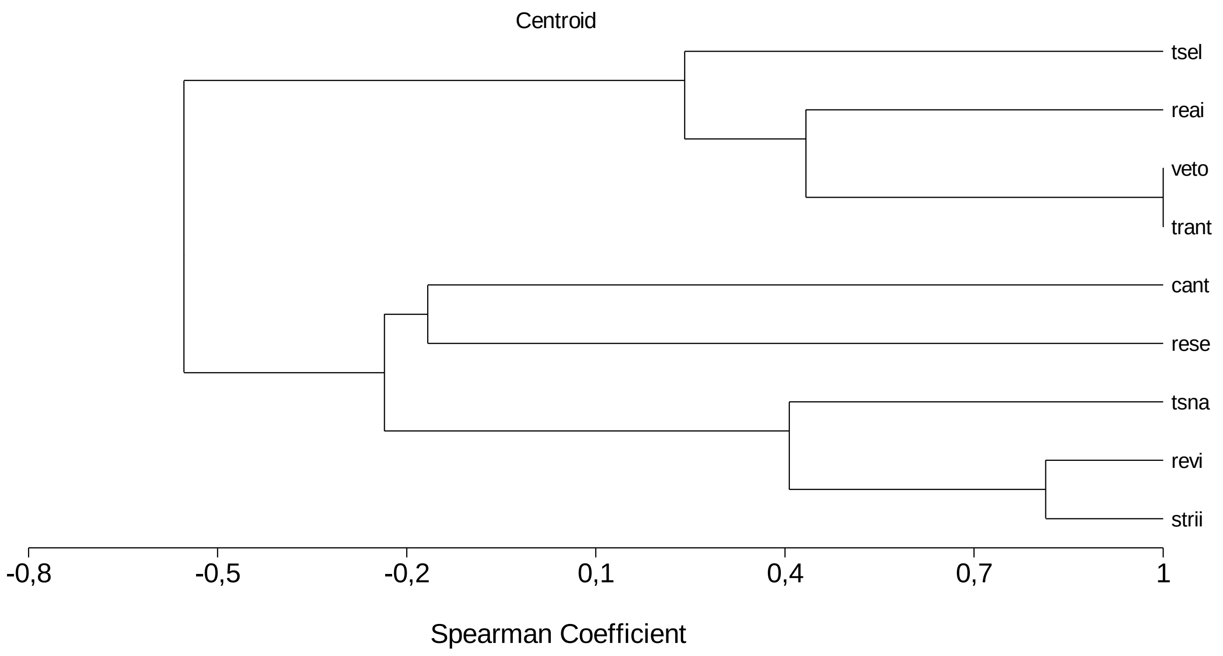

Milk sheep (French Basque region)

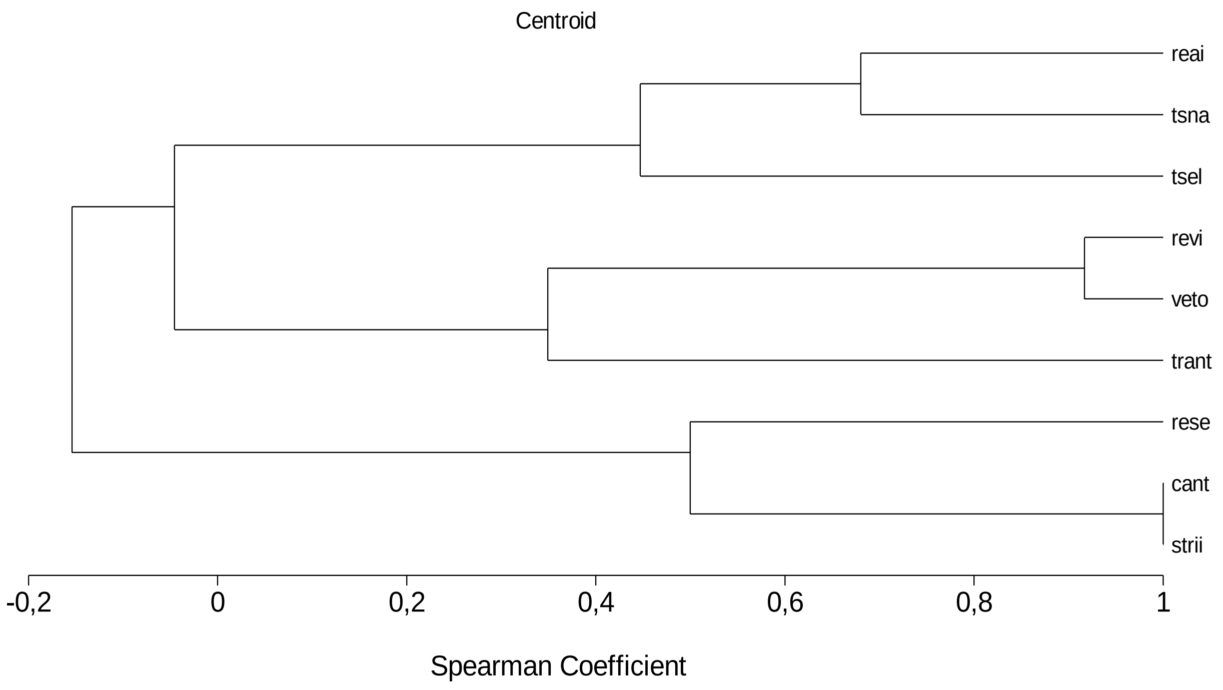

Supplement: Supplementary file 1 [file pathogens-13-00297-s001.zip › pathogens-2885416-supplementary-Figure S1.pdf]
